# Estimating the Causal Impact of Proximity to Gold and Copper Mines on Respiratory Diseases in Chilean Children: An Application of Targeted Maximum Likelihood Estimation

**DOI:** 10.3390/ijerph15010039

**Published:** 2017-12-27

**Authors:** Ronald Herrera, Ursula Berger, Ondine S. von Ehrenstein, Iván Díaz, Stella Huber, Daniel Moraga Muñoz, Katja Radon

**Affiliations:** 1 Occupational and Environmental Epidemiology and NetTeaching Unit, Institute for Occupational, Social and Environmental Medicine, University Hospital Munich (Ludwig Maximilians University), 80336 Munich, Germany; stella.cifuentesbelmar@gmx.de (S.H.); katja.radon@gmail.com (K.R.); 2 Institute for Medical Informatics, Biometry and Epidemiology-IBE, Ludwig Maximilians University, 81377 Munich, Germany; berger@ibe.med.uni-muenchen.de; 3 Departments of Community Health Sciences and Epidemiology, Fielding School of Public Health, University of California Los Angeles, Los Angeles, CA 90025, USA; ovehren@ucla.edu; 4 Department of Biostatistics Bloomberg, School of Public Health, Johns Hopkins University, Baltimore, MD 21218, USA; ildiazm84@gmail.com; 5 Medicine School, Science Faculty, Tarapaca University, Past Staff Catholic University of the North, Coquimbo 1781421, Chile; dmm2640@gmail.com

**Keywords:** children, respiratory health, environmental public health, TMLE, machine learning, causal inference, Chile

## Abstract

In a town located in a desert area of Northern Chile, gold and copper open-pit mining is carried out involving explosive processes. These processes are associated with increased dust exposure, which might affect children’s respiratory health. Therefore, we aimed to quantify the causal attributable risk of living close to the mines on asthma or allergic rhinoconjunctivitis risk burden in children. Data on the prevalence of respiratory diseases and potential confounders were available from a cross-sectional survey carried out in 2009 among 288 (response: 69%) children living in the community. The proximity of the children’s home addresses to the local gold and copper mine was calculated using geographical positioning systems. We applied targeted maximum likelihood estimation to obtain the causal attributable risk (CAR) for asthma, rhinoconjunctivitis and both outcomes combined. Children living more than the first quartile away from the mines were used as the unexposed group. Based on the estimated CAR, a hypothetical intervention in which all children lived at least one quartile away from the copper mine would decrease the risk of rhinoconjunctivitis by 4.7 percentage points (CAR: −4.7; 95% confidence interval (95% CI): −8.4; −0.11); and 4.2 percentage points (CAR: −4.2; 95% CI: −7.9;−0.05) for both outcomes combined. Overall, our results suggest that a hypothetical intervention intended to increase the distance between the place of residence of the highest exposed children would reduce the prevalence of respiratory disease in the community by around four percentage points. This approach could help local policymakers in the development of efficient public health strategies.

## 1. Introduction

Copper mining and gold mining are cornerstones of Chile’s economy. In 2014, Chile contributed to 31% of the world’s production of copper and 2% of the gold production. Open-pit mining is the primary extraction method in Chile (used in up to 91% of the mines) [1] involving explosive procedures and heavy machinery. Environmental impacts of open-pit mining include erosion, biodiversity loss, as well as contamination of the groundwater and pollution of ambient air [2,3].

Children are particularly susceptible to air pollution because they spend more time outdoors and have a higher breathing rate than adults [4,5]. Hence, children living in open-pit mining communities in the U.K. consulted their GPs more frequently for respiratory conditions [6]. Furthermore, a study carried out in a Colombian coal mining region indicated a higher prevalence of asthma among exposed compared to non-exposed children [7]. Epidemiological studies investigating environmental air pollution from other sources, such as petrochemical industry, wood factories or incinerators [8,9,10,11,12], consistently indicated associations with asthma and allergies in children. Most of them used proximity to sources as the exposure surrogate [9,13,14], and some suggested a threshold value, i.e., a distance beyond which no association of the exposure with the respiratory diseases can be detected [8,9,13].

We have previously shown associations between distance to open-pit mines and prevalence of respiratory diseases among children living in a rural community in the desert of Northern Chile, where two open-pit mines (one for gold and one for copper) are located close to the community [14]. Given the dry climate (only 12 days with some rainfall per year), exposure levels might be higher compared to other regions where rain washes out the air pollution every once in a while. Wind is usually light, blowing 32% of the time from the mines to the town (west-south-western, western or south-western wind) [15,16]. In that study, we used Bayesian and parametric models to establish the relation between proximity to mines and respiratory diseases, adjusting for potential confounders. However, these models may lead to biased estimates and incomplete control for confounding [17,18]. Furthermore, we did not evaluate the attributable risk mining has on respiratory disease prevalence in the community’s children.

In order to address the aforementioned limitations, we aimed to estimate the causal attributable risk (CAR) of living closer to the mines on asthma or allergic rhinoconjunctivitis. CAR compares, under assumptions, the absolute change in asthma or rhinoconjunctivitis risk that would have been experienced by the target population under a hypothetical intervention consisting of changing proximity levels to the gold or the copper mine. These estimates are then compared to the currently observed proximity levels.

## 2. Materials and Methods

### 2.1. Study Population and Questionnaire

Data for the analyses were obtained from a questionnaire-based cross-sectional study [14,19]. In brief, the study included 288 children attending 1st–6th grade at two larger elementary schools in the community (417 were invited to participate, response: 69%). Approximately 84% of children living in the community of the age under study attend one of these schools. Information on respiratory outcomes and covariates were obtained using the Spanish version of the International Study on Asthma and Allergies in Childhood (ISAAC) questionnaire [20]. The questionnaire was sent home to be answered by the children’s parents. The project was approved by the Ethics Committee of the University Hospital Munich (Ludwig Maximilians University) and by the Ethics Committee of the Universidad Católica del Norte in Coquimbo, Chile.

### 2.2. Respiratory Outcomes

Asthma was defined as “doctor diagnosed asthma” or “taking asthma medication during the 12 months before the survey.” A child was considered to have rhinoconjunctivitis if one or more of the following nasal symptoms were reported: sneezing, itching, nasal congestion or rhinitis 12 months before the survey and if these symptoms occurred in conjunction with itchy, red and watery eyes [20]. We also created a variable coded as “asthma or rhinoconjunctivitis” to assess the overall impact of hypothetical interventions on respiratory health in the community’s children.

### 2.3. Exposure to Mines

**Figure 1:**
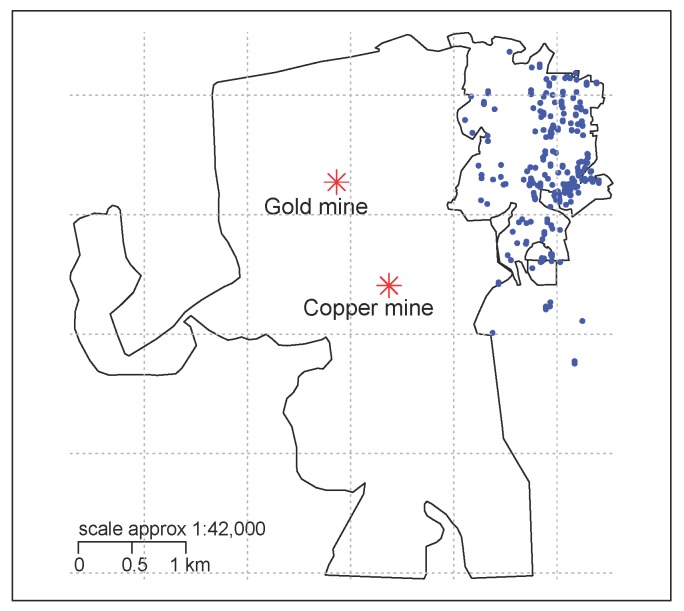
Geographical position of the gold and copper mines and child’s residence. Original positions were changed using jitter to keep confidentiality.

In the absence of available emissions inventory, stationary or personal exposure data, we used proximity to open pit mines as a proxy for exposure. Using the global positioning system (GPS), we established the latitude and longitude coordinates of children’s residences and the primary locations of the mining extraction procedures (Figure 1). Based on this, we calculated residential proximity to the gold and copper mine [14]. We considered the first and the second distance quartile to each mine and to either mine [12] as exposed categories and compared them to those living at least within the median distance.

### 2.4. Potential Confounders

As potential confounders, we included: sex (female vs. male), age (6–7 vs. 8–9, 10–11 and 12 or more years) and family history of atopic disease, i.e., whether a family member ever reported or was diagnosed with one or more of the respiratory diseases under study (no vs. yes). Further, as proxy variables for socioeconomic status (SES), we assessed if the mother (no vs. yes) or father (no vs. yes) of the child worked and whether the child was living with both parents (no vs. yes). We also considered other sources of air pollution, i.e., exposure to cigarette smoking at home (no vs. yes), the energy source for heating used at home (other vs. coal and gas) and type of nearest road (paved vs. dirt road). We used time spent at home (less than 3 vs. 3–6 and more than 6 h/day) and the child’s main place for playing (inside vs. outside) as additional confounders. As a sensitivity analysis, we additionally adjusted the final models for schools.

### 2.5. Statistical Analysis

For our analysis, we used improved methods of causal inference [21,22]. These approaches produce causal attributable risk estimates [23,24], and they are appropriate for cross-sectional study designs [25,26,27,28,29]. Additionally, we used targeted maximum likelihood estimation (TMLE) [22], which is a semi-parametric efficient approach, to estimate the causal attributable risk of asthma or rhinoconjunctivitis. TMLE was implemented jointly with the Super Learner algorithm, a flexible data-adaptive algorithm [30]. Combining TMLE with Super Learner, we were able to improve both the robustness and precision of our estimates.

#### 2.5.1. Parameters of Interest

We aimed to estimate the reduction in the prevalence of respiratory health outcomes (asthma, allergic rhinoconjunctivitis or both) if the study population had been entirely unexposed, i.e., if the whole population lived at least one or two quartiles away from the mines. To quantify this reduction in prevalences in our study population, we estimated the causal attributable risk (CAR). CAR compares the outcome distribution under a hypothetical intervention intended to remove the current exposure in the targeted population, with observed outcome distributions [23,31,32]. Based on the counterfactual framework [21], we were interested in the following causal parameter (Equation (1)):

(1) $$\begin{array}{c} E\left(Y\right)-E\left(Y_{a}\right);a\in A=\left\{1,2\right\} \end{array}$$

where Y represents the observed respiratory disease, being equal to 1 if a child reports having asthma or rhinoconjunctivitis (or both). $Y_{a}$ represents the counterfactual respiratory disease outcome that a child would have if she/he had been exposed to a particular distance quartile, A=a∈A. In our approach, A assumes two possible values, 1 indicating the first distance quartile or 2 representing the median distance to the mines in the studied population. Estimates based on both exposures were compared against the observed prevalences (Equation (1)).

#### 2.5.2. Identification of the Causal Parameter

Once the causal parameters of interest are defined, it is essential to establish some assumptions to identify the parameter from the observed data [33]. First, it was necessary to assume that the observed outcome (Y) that a child experienced under the observed distance quartile to the mine was equal to the counterfactual outcome ($Y_{a}$) under the exposure level, i.e., $Y=Y_{a}$; this is known as the consistency assumption. Second, we assumed that given all used potential confounders (here indicated as W), the potential outcome $Y_{a}$ was independent of the exposure A; i.e., $Y_{a}⫫A|W,\forall a\in A$; this is referred to as the randomization assumption (no unmeasured confounding). Under this assumption, we considered that all measured covariates W were enough to control for confounding the effect of A on Y. Finally, we relied on the positivity assumption, which implies that there was a positive probability for receiving each quartile of exposure A within every combination of covariates among the studied population, also known as the experimental treatment assumption (ETA). The first and second assumptions are untestable with the data [33].

Under these three assumptions, we can express the target parameter of interest regarding the observed data as (Equation (2)):

(2) $$\begin{array}{c} E\left(Y\right)-E\left(Y_{a}\right)=E\left(Y\right)-E_{w}\left\{E\left(Y|A=a,W\right)\right\};a\in A=\left\{1,2\right\} \end{array}$$

The equality in Equation (2) is a function of the observed data distribution, and it represents the statistical estimand.

#### 2.5.3. Estimation of Parameters of Interest

From Equation (2), the first term, E(Y), can be estimated using the prevalence of each respiratory outcome, i.e., the proportion of children with asthma or allergic rhinoconjunctivitis. The counterfactual part of the target parameter in Equation (2), $E_{w}\left\{E\left(Y|A=a,W\right)\right\}$, was estimated using targeted maximum likelihood estimation (TMLE) [22,33]. TMLE is a two-step method: first, an estimation of the conditional expectation of the outcome given the exposure and covariates is obtained, E(Y|A,W). Then, using these estimates, a second step occurs, the bias-reduction step, where the initial estimate of the outcome regression is updated using the estimation of the exposure mechanism, P(A=a|W);a∈A={1,2} (exposure probabilities), and it is referenced as the “targeting” step [33]. In the last step, the updated estimation of E(Y|A,W) is used to estimate the parameter in Equation (2). TMLE has the property of “double robustness,” meaning that the estimated parameters will be consistent if either one of the two initial estimators is consistent, and TMLE estimates will reach the lowest asymptotic variance among the reasonable estimators (efficiency) if both are estimated consistently. We estimated the exposure probabilities, as well as the outcome regression using an ensemble predictor known as Super Learner [30]. Super Learner constructs a convex combination of candidate predictors in a user-given library. Subsequently, weights are chosen to minimize the cross-validated log-likelihood of the resulting ensemble. As using regressions to calculate the final estimator could generate a bias increase [34], we corrected this issue using the Super Learner algorithm.

#### 2.5.4. Missing Values

Several covariates of interest had missing values (Table 1). Therefore, we used multiple imputation procedures on all confounders with missing values, but not so for the outcome variables, nor the exposure variables. During imputation, we created five imputed datasets using the R library *mice* [35]. With each one of the five imputed datasets, we estimated a CAR, and results from these five parameters were used to calculate a combined CAR estimate with the respective confidence intervals using Rubin’s rules [36]. Analyses were performed in the R (Version 3.4.1, R Foundation for Statistical Computing, Viena, Austria) programming language [37] using the tmle [38] and SuperLearner [39] packages.

## 3. Results

### 3.1. Descriptive Results

Of the 288 participating children, we excluded three children for having missing residence locations and ten children who lived in isolated zones from the community. Therefore, the analysis included 275 children living in the community.

The point prevalence was 24% for asthma, 34% for rhinoconjunctivitis and 44% for asthma and rhinoconjunctivitis combined. The mean distance to the mines was 2.08 km (standard deviation (SD): 0.26; range 1.33–2.77 km) for the gold mine and 1.95 km (SD: 0.37; range 0.87–3.11 km) for the copper mine. Children living within the first quartile of distance to the mines reported the highest prevalence of respiratory symptoms. Using the median as the cut-off point, no statistically relevant differences in respiratory diseases were found for the resulting two exposure groups (Table 2).

Forty-six percent of the included participants were female; mean age was 9.05 years (SD: 1.88 years, range 6–15 years). Two-thirds of the children lived with both parents. In 84% of the families, the father was working, while in 25%, the mother was holding a job. Children whose father worked were more likely to live further away from the mines than those whose father did not work (Table 1). The proportion of parents with atopic diseases was 31%. Children spending fewer hours at home also lived further away from the mines (Table 1).

### 3.2. Causal Attributable Risk

Based on the estimated CARs, a hypothetical intervention in which all children lived further away than the first quartile of distance to the copper mine would decrease the risk of allergic rhinoconjunctivitis by 4.1 percentage points (CAR: −4.1%, 95% confidence interval: −6.9; −1.3%) and by 2.9 percentage points for both respiratory outcomes combined (CAR: −2.9%, 95% CI: −5.7%; −0.1%). Estimates were similar for distance to either mine and rhinoconjunctivitis (CAR: −4.7%, 95% CI: −8.4; −1.1%) and asthma and rhinoconjunctivitis combined (CAR: −4.2%, 95% CI: −7.9; −0.5%). However, no association was seen for asthma, distance to the gold mine and using the median as the cut-off (Table 3).

Adjusting for schools, results remained robust for all outcomes with respect to the distance to the copper and the gold mine. However, using the first distance quartile to either mine as exposure variable, estimates lost statistical significance (Appendix A, Table A1).

## 4. Discussion

Using a semi-parametric targeted approach, we were able to estimate CAR from proximity to mining industries among children living in the surroundings of open-pit copper and gold mines in Northern Chile. Results indicated that a hypothetical intervention intending to increase the distance from children’s home to the mines could result in a reduction of rhinoconjunctivitis prevalence in the studied population by up to 4.7 percentage points (95% CI: −8.4%; −1.1%). Thereby, our method estimates the public health impact of such an intervention, which could not be done using the standard statistical approaches (e.g., logistic regression estimates).

**Figure A1:**
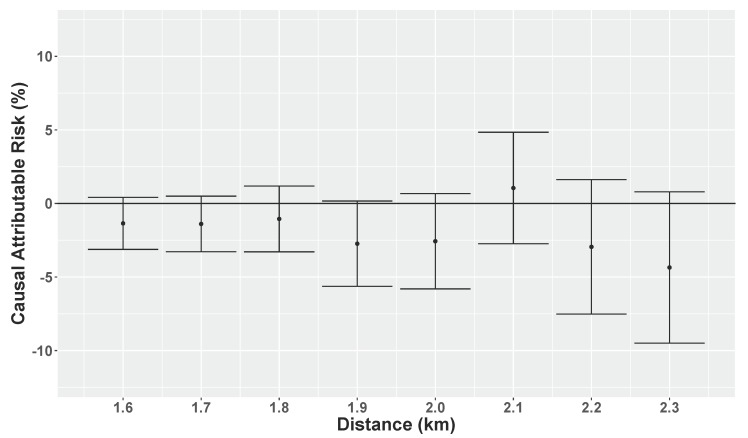
Estimated CAR of asthma in children, expressed as percentage points with whiskers indicate 95% confidence intervals and different distances to the gold mine. Data from children living in a rural mining community in the north of Chile, 2009.

**Figure A2:**
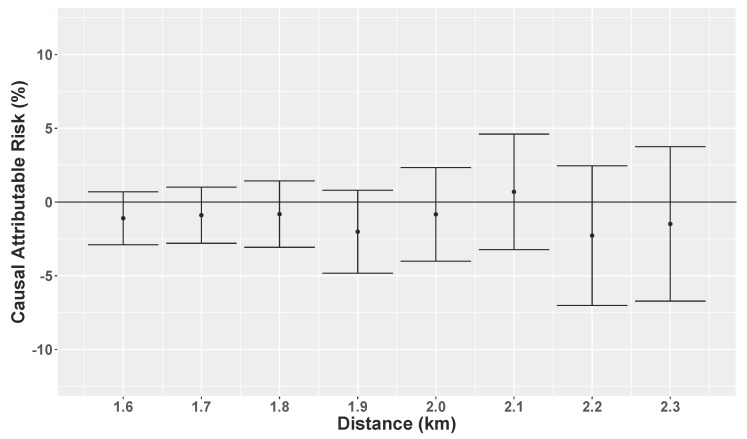
Estimated CAR of rhinoconjunctivitis in children, expressed as percentage points with whiskers indicate 95% confidence intervals and different distances to the gold mine. Data from children living in a rural mining community in the north of Chile, 2009.

**Figure A3:**
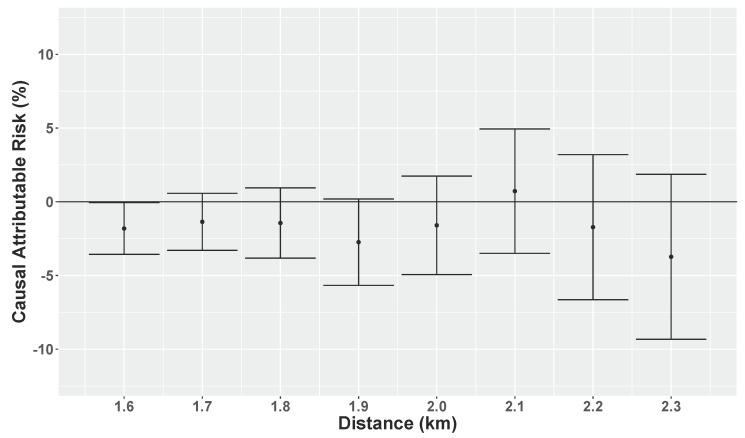
Estimated CAR of asthma or rhinoconjunctivitis in children, expressed as percentage points with whiskers indicate 95% confidence intervals and different distances to the gold mine. Data from children living in a rural mining community in the north of Chile, 2009.

**Figure A4:**
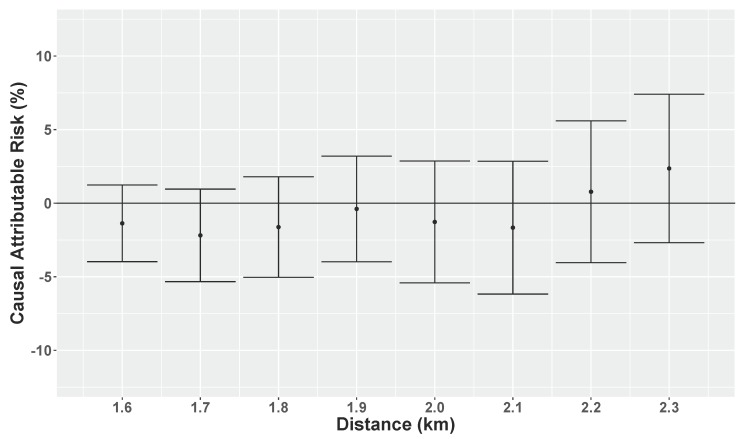
Estimated CAR of asthma in children, expressed as percentage points with whiskers indicate 95% confidence intervals and different distances to the copper mine. Data from children living in a rural mining community in the north of Chile, 2009.

**Figure A5:**
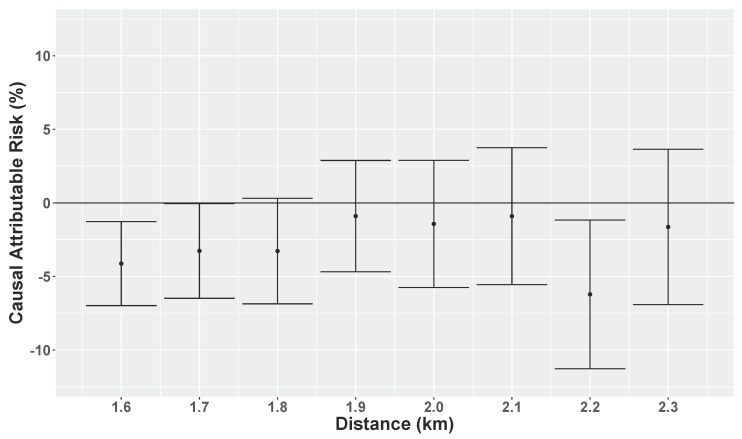
Estimated CAR of rhinoconjunctivitis in children, expressed as percentage points with whiskers indicate 95% confidence intervals and different distances to the copper mine. Data from children living in a rural mining community in the north of Chile, 2009.

**Figure A6:**
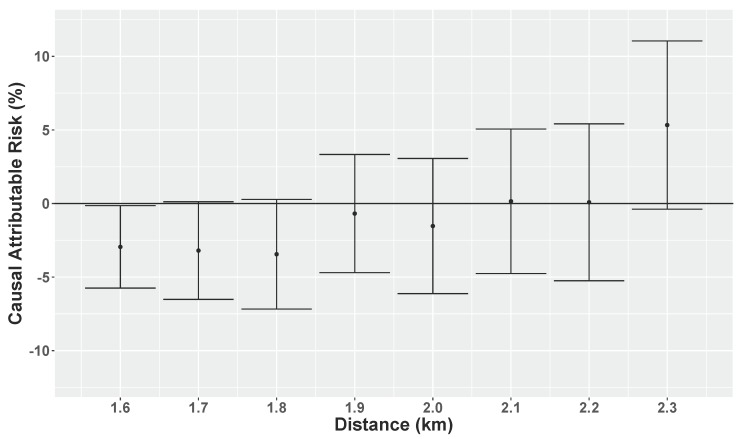
Estimated CAR of asthma or rhinoconjunctivitis in children, expressed as percentage points with whiskers indicate 95% confidence intervals and different distances to the copper mine. Data from children living in a rural mining community in the north of Chile, 2009.

With the standard approach, we previously found the minimum distance between the mines and address of the children associated with increased odds of respiratory diseases. These previous results are not directly comparable with the results presented here because they were based on Bayesian and parametric models. Nevertheless, our new findings strengthen the conclusions from the earlier study. Based on the previous results, we dichotomized our exposure [14]. Additionally, we examined the nonlinearity of the exposure-response association plotting the distances between the place of residence and the mines (Appendix A, Figure A1, Figure A2, Figure A3, Figure A4, Figure A5 and Figure A6). While the associations were linear for the distance to the gold mine (Figure A1, Figure A2 and Figure A3), a U-shape association was found for the distance to the copper mine (Figure A4, Figure A5 and Figure A6). Understanding the shape of the association needs to be taken into account when deciding upon public health interventions [40].

Our approach has several strengths. First, we used the distance between the place of residence and the mines as a proxy for ambient dust exposure, which could be a good surrogate measure of long-term exposure when exposure monitoring sites are missing [9]. Secondly, there was only light traffic in the area and other related outdoor air pollutants. Therefore, the described association is not expected to be confounded by other sources [5,8,41]. We controlled for several potential confounders, including SES and other indoor air pollutants (e.g., second-hand smoke). Another source of indoor exposure, gas cooking, was not considered as 95% of the population used gas for cooking. Our results were robust when adjusting for a number of potential confounders. One may argue that kids spent much of their time in schools and therefore, associations might be confounded by school. However, our sensitivity analyses did not confirm this hypothesis. Our questionnaire instrument was validated for a worldwide study [20] and is thus expected to estimate the prevalence of the outcome correctly. To address the challenge of missing data and relatively small sample size, we used multiple imputation methodologies to use all possible information from the study. Using a counterfactual approach, we were able to estimate CARs to assess the effect of a potential public health intervention, which might be helpful for policymakers [18,42,43]. This methodology has not yet been used to study exposure to open-pit mining and respiratory health. In our estimations, we used TMLE, which is doubly robust to model misspecification. Lastly, combining TMLE with the Super Learner algorithm helped us to correct parametric misspecification bias and thereby guaranteed accurate inference for the TMLE method [33].

Several limitations need to be considered when interpreting our findings. Despite using multiple imputation procedures to handle missing values of the covariates, we lost 17% of the sample because of missing data in the outcome variables. Moreover, our cross-sectional design does not permit assessing the time sequence of exposure and outcome [44]. However, we believe that this condition was met in our study since 98% of the children were born in the community (data were not shown), and the open-pit mining procedures started in the early 1990s, so reverse causation is unlikely an issue for our study. Another consideration is the experimental treatment assumption (ETA). It is possible that we did not record some characteristics of the children or the neighborhood. However, parameter estimates using targeted maximum likelihood estimation are robust to possible ETA violations [23,45]. Possible ETA violations must be investigated to guarantee the causal interpretation of the parameter estimate, especially when transferring our findings based on CARs to other populations [23,46]. Nevertheless, we assume that we had enough variability within each quartile of the proximity to the mines, various covariate strata and sample size, hence the positivity assumption is reasonable.

## 5. Conclusions

Our research was one of the first studies in Latin America assessing the causal impact of open-pit mining on respiratory diseases. We found that living close to open-pit mines could increase the respiratory disease burden in children living in a Northern Chile community. As it is not possible to change the location of the mines (mines must be where the ores are located), a relocation of the population would be a suggested policy intervention. Whether this costly approach is efficient and agreeable to the population needs further evaluation.

## Figures and Tables

**Table 1:** Socio-demographics by living distances to a gold and a copper mine among 275 children living in a rural mining community in the north of Chile, 2009.

Distance to		Gold Mine > Quartile 1		Copper Mine > Quartile 1		Either Mine ${}^{†}$ > Quartile 1	
	Total *n* per Category	%	(*n*)	%	(*n*)	%	(*n*)
**Sex**							
Female	(126)	75	(94)	75	(94)	75	(94)
Male	(149)	75	(112)	75	(112)	74	(111)
**Age**							
6–7 years	(74)	77	(57)	76	(56)	77	(57)
8–9 years	(86)	74	(64)	76	(65)	76	(65)
10–11 years	(82)	70	(57)	72	(59)	70	(57)
≥12 years	(33)	85	(28)	79	(26)	79	(26)
**Living with both parents (NA = 9)**							
No	(81)	72	(58)	68	(55)	70	(57)
Yes	(185)	76	(140)	78	(144)	76	(141)
**Parental atopic disease (NA = 30)**							
No	(160)	77	(123)	77	(123)	76	(122)
Yes	(85)	73	(62)	76	(65)	76	(65)
**Mother working (NA = 15)**							
No	(191)	76	(146)	74	(142)	74	(142)
Yes	(69)	70	(48)	78	(54)	75	(52)
**Father working (NA = 22)**							
No	(22)	68	(15)	64	(14)	54	(12)
Yes	(231)	78	(181)	78	(181)	79	(182)
**Hours child stay at home (NA = 61)**							
Less 3 h	(20)	95	(19)	85	(17)	85	(17)
3–6 h	(46)	74	(34)	72	(33)	74	(34)
More than 6 h	(148)	71	(105)	76	(112)	73	(108)
**Place child play most of the time (NA = 9)**							
Inside	(105)	77	(81)	75	(79)	78	(82)
Outside	(161)	73	(118)	75	(120)	72	(116)
**Smoking in child’s presence (NA = 28)**							
No	(180)	77	(139)	73	(132)	74	(134)
Yes	(67)	69	(46)	81	(54)	76	(51)
**Nearest road (NA = 10)**							
Dirt	(57)	67	(38)	60	(34)	58	(33)
Paved	(208)	77	(160)	79	(164)	79	(164)
**Type of heater (NA = 69)**							
Other	(80)	78	(62)	78	(62)	76	(61)
Coal and Gas	(126)	73	(92)	72	(91)	73	(92)

^†^ Indicates distances above the first quartile to either mine; NA: missing values.

**Table 2:** Prevalence and crude odds ratios of respiratory diseases by living distances to gold and copper mines among 275 children living in a rural mining community in the north of Chile, 2009.

Distance to			Asthma			Rhinoconjunctivitis			Asthma or Rhinoconjunctivitis		
		Overall Prevalence	24% (n=66)			34% (n=93)			44% (n=121)		
		Missing Data	(NA = 35)			(NA = 17)			(NA = 26)		
		Total *n* per Category	%	(*n*)	OR ${}^{a}$ (95% CI)	%	(*n*)	OR ${}^{a}$ (95% CI)	%	(*n*)	OR ${}^{a}$ (95% CI)
**Gold mine**											
**Quartile 1 (1.9 km)**	≤	(69)	32	(22)	1.67 (0.89; 3.11)	41	(28)	1.54 (0.86; 2.75)	52	(36)	1.58 (0.89; 2.84)
	>	(206)	21	(44)	1	32	(65)	1	41	(85)	1
**Quartile 2 (2.3 km)**	≤	(138)	24	(34)	1.01 (0.58; 1.79)	35	(48)	1.08 (0.65; 1.80)	44	(61)	0.99 (0.60; 1.62)
	>	(137)	23	(32)	1	33	(45)	1	44	(60)	1
**Copper mine**											
**Quartile 1 (1.6 km)**	≤	(69)	30	(21)	1.47 (0.78; 2.72)	45	(31)	1.79 (1.01; 3.16)	57	(39)	1.70 (0.97; 3.01)
	>	(206)	22	(45)	1	30	(62)	1	39	(82)	1
**Quartile 2 (2.0 km)**	≤	(138)	25	(35)	1.18 (0.67; 2.09)	38	(52)	1.38 (0.83; 2.31)	47	(65)	1.27 (0.78; 2.10)
	>	(137)	23	(31)	1	30	(41)	1	41	(56)	1
**Both mines**											
**Quartile 1** ${}^{†}$	≤	(96)	30	(29)	1.61 (0.90; 2.87)	43	(41)	1.81 (1.07; 3.08)	53	(51)	1.66 (0.99; 2.81)
	≥	(179)	20	(37)	1	30	(52)	1	40	(70)	1
**Quartile 2** ${}^{‡}$	≤	(101)	24	(41)	0.79 (0.44; 1.41)	35	(61)	0.75 (0.45; 1.27)	44	(76)	0.74 (0.44; 1.24)
	>	(174)	25	(25)	1	32	(32)	1	45	(45)	1

${}^{†}$ Indicates distances above the first quartile to either mine; ${}^{‡}$ indicates distances above the second quartile to either mine; NA: missing values; ${}^{a}$ unadjusted odds ratios using logistic regression.

**Table 3:** Estimated causal attributable risk (CAR) of respiratory diseases in children, expressed as percentage points with the 95% confidence intervals (95% CI). Data from children living in a rural mining community in the north of Chile, 2009.

		Asthma n=240 ^*a*^		Rhinoconjunctivitis n=262 ^*a*^		Asthma or Rhinoconjunctivitis n=229 ^*a*^	
		CAR ${}^{b}$	95% CI	CAR ${}^{b}$	95% CI	CAR ${}^{b}$	95% CI
**Gold mine**	**Quartile 1 (1.9 km)**	−2.7%	(−5.7%; 0.3%)	−2.1%	(−4.8%; 0.8%)	2.7%	(−5.7%; 0.2%)
	**Quartile 2 (2.3 km)**	−4.3%	(−9.5%; 0.8%)	−1.5%	(−6.7%; 3.4%)	3.7%	(−9.3%; 1.9%)
**Copper mine**	**Quartile 1 (1.6 km)**	−1.4%	(−4.3%; 1.6%)	−4.1%	(−6.9%; −1.3%)	−2.9%	(−5.7%;−0.1%)
	**Quartile 2 (2.0 km)**	−1.3%	(−5.4%; 2.9%)	−1.4%	(−5.7%; 2.9%)	−1.5%	(−6.1%; 3.1%)
**Either mine**	**Quartile 1**	−3.5%	(−7.1%; 0.1%)	−4.7%	(−8.4%; −1.1%)	−4.2%	(−7.9%; −0.5%)
	**Quartile 2**	0.5%	(−3.9%; 5.0%)	−2.0%	(−6.7%; 2.7%)	0.7%	(*C*%; 5.6%)

${}^{a}$ Total sample used in the estimation after multiple imputations of the covariates; ${}^{b}$ negative values imply a reduction in prevalences due to a hypothetical intervention.

## Abbreviations

The following abbreviations are used in this manuscript:

CAR	Causal attributable risk
CI	Confidence interval
ETA	Experimental treatment assumption
GPS	Global positioning system
GP	General practitioners
ISAAC	International Study on Asthma and Allergies in Childhood
SES	Socioeconomic status
TMLE	Targeted maximum likelihood estimation

## Appendix A.

### Tables

**Table A1:** Estimated causal attributable risk (CAR) of respiratory diseases in children, expressed as percentage points with the 95% confidence intervals (95% CI) including schools as a potential confounder. Data from children living in a rural mining community in the north of Chile, 2009.

		Asthma n=240 ^*a*^		Rhinoconjunctivitis n=262 ^*a*^		Asthma or Rhinoconjunctivitis n=229 ^*a*^	
		CAR ${}^{b}$	95% CI	CAR ${}^{b}$	95% CI	CAR ${}^{b}$	95% CI
**Gold mine**	**Quartile 1 (1.9 km)**	−2.8%	(−6.0%; 0.3%)	−2.4%	(−5.4%; 0.6%)	−3.0%	(−6.8%; 0.2%)
	**Quartile 2 (2.3 km)**	−0.4%	(−4.5%; 3.6%)	−1.2%	(−5.4%; 2.9%)	−0.3%	(−4.7%; 4.1%)
**Copper mine**	**Quartile 1 (1.6 km)**	−1.3%	(−4.3%; 1.2%)	−3.6%	(−6.7%; −0.4%)	−3.2%	(−6.5%; −0.01%)
	**Quartile 2 (2.0 km)**	−1.6%	(−5.7%; 2.4%)	−3.7%	(−8.0%; 0.6%)	−3.2%	(−7.6%; 1.2%)
**Either mine**	**Quartile 1**	−1.2%	(−3.7%; 1.3%)	−1.6%	(−4.0%; 0.9%)	−2.0%	(−4.5%; 0.5%)
	**Quartile 2**	0.9%	(−3.6%; 5.4%)	−1.7%	(−6.4%; 3.0%)	1.6%	(−3.3%; 6.6%)

*^a^* Total sample used in the estimation after multiple imputations of the covariates; *^b^* negative values imply a reduction in prevalences due to a hypothetical intervention.
